# Comparative evaluation of linear and exponential amplification techniques for expression profiling at the single-cell level

**DOI:** 10.1186/gb-2006-7-3-r18

**Published:** 2006-03-07

**Authors:** Tatiana Subkhankulova, Frederick J Livesey

**Affiliations:** 1 Gurdon Institute and Department of Biochemistry, University of Cambridge, Tennis Court Road, Cambridge, CB2 1QN, UK

## Abstract

**Background:**

Single-cell microarray expression profiling requires 10^8^-10^9^-fold amplification of the picogram amounts of total RNA typically found in eukaryotic cells. Several methods for RNA amplification are in general use, but little consideration has been given to the comparative analysis of those methods in terms of the overall validity of the data generated when amplifying from single-cell amounts of RNA, rather than their empirical performance in single studies.

**Results:**

We tested the performance of three methods for amplifying single-cell amounts of RNA under ideal conditions: T7-based *in vitro *transcription; switching mechanism at 5' end of RNA template (SMART) PCR amplification; and global PCR amplification. All methods introduced amplification-dependent noise when mRNA was amplified 10^8^-fold, compared with data from unamplified cDNA. PCR-amplified cDNA demonstrated the smallest number of differences between two parallel replicate samples and the best correlation between independent amplifications from the same cell type, with SMART outperforming global PCR amplification. SMART had the highest true-positive rate and the lowest false-positive rate when comparing expression between two different cell types, but had the lowest absolute discovery rate of all three methods. Direct comparison of the performance of SMART and global PCR amplification on single-cell amounts of total RNA and on single neural stem cells confirmed these findings.

**Conclusion:**

Under the conditions tested, PCR amplification was more reliable than linear amplification for detecting true expression differences between samples. SMART amplification had a higher true-positive rate than global amplification, but at the expense of a considerably lower absolute discovery rate and a systematic compression of observed expression ratios.

## Abstract 2

Comparison of the performance of three methods for amplifying single-cell amounts of RNA for use in expression profiling shows that PCT amplification is more reliable than linear amplification.

## Background

Whole-genome expression profiling has many applications in areas of research where acquisition of small, highly specific tissue or cell samples is required for accurate expression analysis, such as oncology, neuroscience and development biology [1,2]. The application of array technology or sequencing-based expression profiling technologies, such as SAGE [3], to single cells, however, requires either a considerable increase in the sensitivity of those assays or the amplification of the input RNA [4]. Amplification of the starting RNA population is in common use to generate labeled microarray targets from limiting amounts of RNA. Commonly used amplification techniques are based on two different approaches: linear isothermal amplification by *in vitro *transcription (IVT) of the cDNA population into labeled complementary RNA (cRNA), typically using T7 RNA polymerase [5,6], and PCR amplification of the entire population of cDNA following reverse transcription [7-9].

The most commonly used mechanism for linear isothermal RNA amplification is based on T7 RNA polymerase-mediated IVT [6]. Several protocols based on this technique have been developed and used for microarray analysis [10-12]. Linear isothermal RNA amplification can increase the starting amounts of mRNA up to 1,000-fold in one round, while second or possibly third rounds of amplification are possible [6,13]. Amplified RNA (aRNA) samples have been shown to generate reproducible microarray data when compared with non-amplified mRNA and closely approximate original samples [13-15]. It has been found, however, that the resulting microarray data can vary depending on details of the amplification protocol, including the amount of starting material [13], whether antisense or sense RNA is produced [16], and the number of rounds of amplification performed. In addition, time-dependent RNA degradation during IVT can introduce noise to the resulting microarray data [17].

Several PCR-based methods of RNA amplification have been developed as an alternative to linear IVT-based techniques. These include global PCR amplification following polyadenylation, which we shall call global amplification (GA) [18], 3' end amplification (TPEA) [9] and strand-switching-mediated reverse transcription amplification, commonly known as switching mechanism at 5' end of RNA template (SMART) [19,20]. PCR has a number of potential advantages over linear isothermal amplification: it is faster, more cost effective, with an almost unlimited degree of amplification [18,21,22]. The disadvantage of relatively simple PCR-based exponential amplification is a general assumption that it introduces unacceptable biases to microarray data.

A key development in single-cell PCR amplification was the introduction of a strategy to normalize the size distribution of the resulting cDNA fragments such that the range of cDNA lengths falls between several hundred and a thousand bases [7,8]. This was achieved by restricting the initial reverse transcription to the most 3' sequence by limiting deoxyribonucleotide concentrations and the time of reaction [18]. Global cDNA amplification with this method enables amplification of picograms of mRNA with preservation of relative abundance of cDNAs through amplification as high as 10^11^-fold under ideal conditions [18].

Previously we found that SMART-amplified cDNA results in a systematic underestimation of the magnitude of gene-expression differences between samples when amplifying microgram amounts of total RNA [23]. Subsequent work has also found that SMART cDNA generates reproducible data while introducing systematic changes in gene-expression ratios compared to those observed from unamplified material [21,22,24]. The performance of SMART in amplifying single-cell amounts of RNA has not been investigated, however, nor has it been compared to other methods for single-cell RNA amplification.

To date, little consideration has been given to direct comparison of existing methods of mRNA amplification in side-by-side experiments, particularly within the same range of amplification. In this study we investigated whether linear and exponential techniques for amplifying single-cell equivalents of total RNA introduce biases to microarray data, the nature of those biases and the levels of noise for each particular amplification method. Our goal was to define which technique is more acceptable for picogram-level expression profiling.

To do so, we analyzed the reproducibility of each method, in terms of the errors each method introduced into the amplified cDNA population, and how each method performed in identifying truly differentially expressed genes while minimizing the rate of false positives in the resulting datasets. To estimate this, we compared data generated using each method with data generated from unamplified RNA from the same sources. The three methods we studied were T7-based *in vitro *amplification (IVTIII), and two PCR-based methods, SMART and GA.

Overall, we found that under the conditions tested of amplifying picogram quantities of total RNA, PCR amplification outperformed IVT in several key areas. The two PCR-based methods were found to have complementary advantages: SMART had a high true-discovery rate but a low absolute number of differentially expressed genes, whereas GA identified the largest number of true positives at the expense of a considerably higher false-positive rate. An analysis of the performance of the two PCR-based methods in generating data from single-cell equivalents of total RNA and from single mammalian neural stem cells confirmed those findings.

## Results

### Experimental design and yields of amplified DNA/cRNA

The objective of this study was to compare amplification techniques and choose the most reliable method for single-cell expression profiling. Therefore, all methods were tested using single-cell amounts of total RNA. It has been estimated that a single cell contains approximately 0.1 pg of mRNA or 10 pg of total RNA. This amount would need to be amplified 10^8^-10^9^-fold to generate enough DNA/RNA targets for hybridization on two-color microarrays. Dilution of a complex population of total RNA down to low concentrations can, however, cause a sampling effect, resulting in random representation of different species of mRNA in each aliquot.

To minimize this source of error we took a relatively high amount, 10 ng of total RNA, for the initial reverse transcription reaction for each of the PCR amplification methods assessed: GA [7,8] standard SMART [25] and modified SMART (SM37) One-fifth of the reversed transcribed cDNA was used for the initial ten cycles of PCR amplification for both SMART and GA, after which 1/200 of that PCR product was used for a further 28 cycles of PCR amplification.

**Figure 1:**
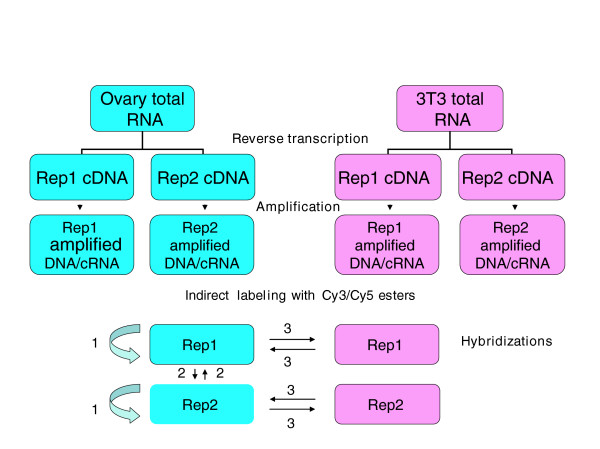
Experimental design for testing RNA amplification techniques. The scheme for estimating errors and testing the performance of each amplification technique in generating microarray expression data is shown. Total RNA was isolated from two mouse cell lines, an ovary epithelial cell line (OV) and 3T3 fibroblasts (3T3). Unamplified targets were synthesized from 100 μg of total RNA by reverse transcription. RNA was amplified by using linear T7 based- or exponential PCR-based amplification (see text for details). Synthesized DNA/cRNA samples were indirectly labeled with Cy3/Cy5 fluors. Labeled targets were co-hybridized on oligonucleotide arrays (see Materials and methods for details). Design 1, same vs same control hybridization. Labeled DNA/cRNA were divided in two parts and each was coupled with either Cy3 or Cy5 NHS-esters followed by co-hybridization on the same slide. Design 2, hybridization of technical replicates. Pairs of technical replicates synthesized from ovarian cell line total RNA by each amplification method or by reverse transcription (unamplified cDNA) were compared. Design 3, comparison of gene expression between two different cell lines by hybridization of cDNAs amplified by the same technique.

Similarly, 10 ng of total test RNA were taken for the first round of T7-based linear amplification, 1/10 of the first-round amplified cRNA was used for the second round of amplification and 1/100 of the second round amplified cRNA was used for the third round of amplification. Typically 15-20 mg of amplified cDNA and 25-50 mg of cRNA from the third round were obtained from these procedures. Both amplified cDNA and cRNA were indirectly labeled with Cy3/Cy5 dyes, as was unamplified cDNA. Labeled samples were combined and co-hybridized on oligonucleotide microarrays using the scheme shown (Figure 1). This scheme was designed to identify several potential sources of error in the microarray analysis of amplified cDNA, including platform-dependent errors (hybridization-dependent errors) and systematic biases introduced by each amplification method.

### Indirect labeling of cDNA or cRNA with Cy3 or Cy5 did not introduce a bias to microarray data

To estimate the contribution of labeling to microarray data noise, we performed a set of self-self hybridizations (Figure 1): both unamplified or amplified aminoallyl-labeled DNA/cRNA were divided into two parts and each was coupled with either Cy3 or Cy5 NHS-esters followed by co-hybridization on the same slide.

In microarray analysis, data are frequently represented by the MA plot [26]. This data representation plots gene-expression log ratios, log_2_(*R*/*G*) or *M *values, against the log mean intensities, *log*_2_ or *A *values, where *R *and *G *represent the Cy3 and Cy5 intensities for a given spot. If we assume that *d*_1 _= log_2_*R *and *d*_2 _= log_2_*G*, then

*M *= log_2_ = *d*_1 _- *d*_2_

*A *= log_2_ = 1/2log_2 _(*R·G*) = 1/2(*d*_1 _+ *d*_2_)

Thus, an MA plot for two technical replicates is the same as the Bland-Altman plot for two measurements [27]: the *x *axis shows the mean of the results of the two measurements ([*d*_1 _+ *d*_2_]/2), whereas the *y *axis represents the absolute difference between the measurements ([*d*_1 _- *d*_2_]). The Bland-Altman plot may also be used to assess the repeatability of a method by comparing repeated measurements using one single method on a series of subjects. The coefficient of repeatability (CR) can be calculated as 1.96 (or 2) times the standard deviations of the differences between the two measurements (*d*_2 _and *d*_1_) [27].



**Figure 2:**
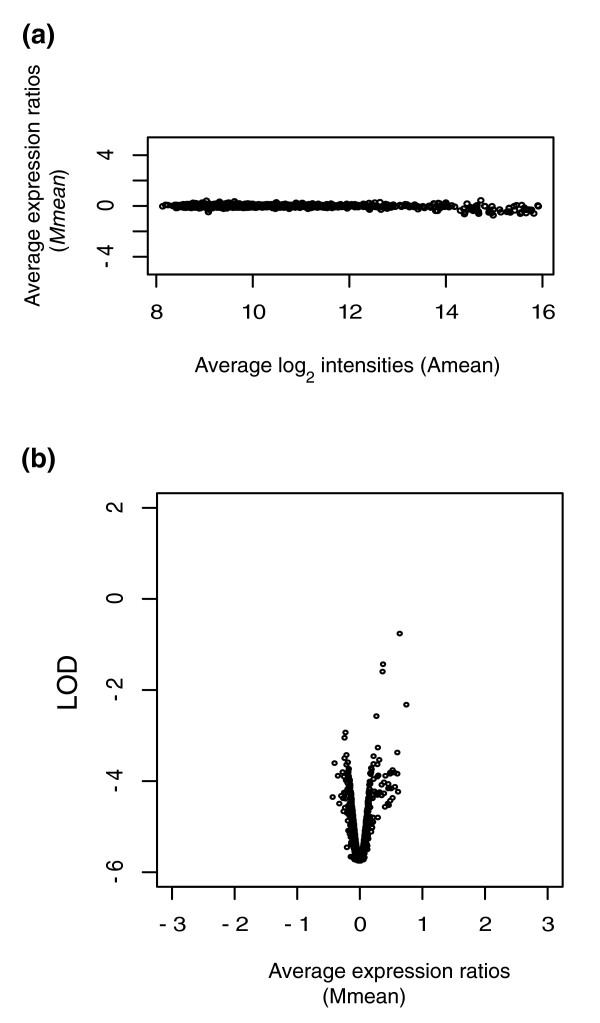
Indirect labeling of microarray targets with either Cy3 or Cy5 dyes introduced insignificant levels of noise to microarray data. **(a) **MA plot for averaged microarray data obtained in two dye-swapped, same vs same target hybridizations. An MA plot plots log-ratio *R *vs *G *against the overall intensity of each spot (see text for explanation). The log-ratio is represented by the *M *value, *M *= log_2_(*R*) - log_2_(*G*), and the overall intensity by the A value, *A *= (log_2_(*R*) + log_2_(*G*))/2. Targets were obtained by three rounds of T7-amplification (IVTIII). Amean and Mmean were calculated as the averages of *M *or *A *values for two microarray experiments and plotted. **(b) **A volcano plot shows the likelihood of expression difference (LOD) for genes in these two hybridizations selected by an empirical Bayesian method (see Materials and methods for details). The volcano plot shows the relationship between the log_2_-fold change in expression levels and the LOD to assess the significance of the differences between two targets. There are no genes with LOD greater than zero and thus there are no significant differences between Cy3- and Cy5-labeled targets.

Thus, if two technical replicates generated by any amplification method are co-hybridized on the same microarray slide, the MA plot is a measure of the repeatability of the method and the coefficient of repeatability can be calculated as two standard deviations of *M *values. A typical MA plot for self-self hybridized targets is shown (Figure 2). Most genes should not be found to be differentially expressed in this comparison, and, as predicted, the majority of points are in a cloud around *M *= 0.

Regardless of the type of amplification, CR values for same versus same hybridizations were low, and considerably smaller than if two independent replicate samples or two different samples were co-hybridized (Table 1). In addition, there was no correlation for paired dye-swap self-self hybridizations (Pearson's correlation coefficients were 0.005-0.2) as well as no outliers selected by an empirical Bayesian method with threshold at likelihood of expression difference (LOD) score of zero or higher (Figure 2). Therefore, the levels of noise introduced by the labeling procedure were relatively low and are unlikely to occur because of dye bias.

### All methods of RNA amplification introduce errors to microarray data

**Figure 3:**
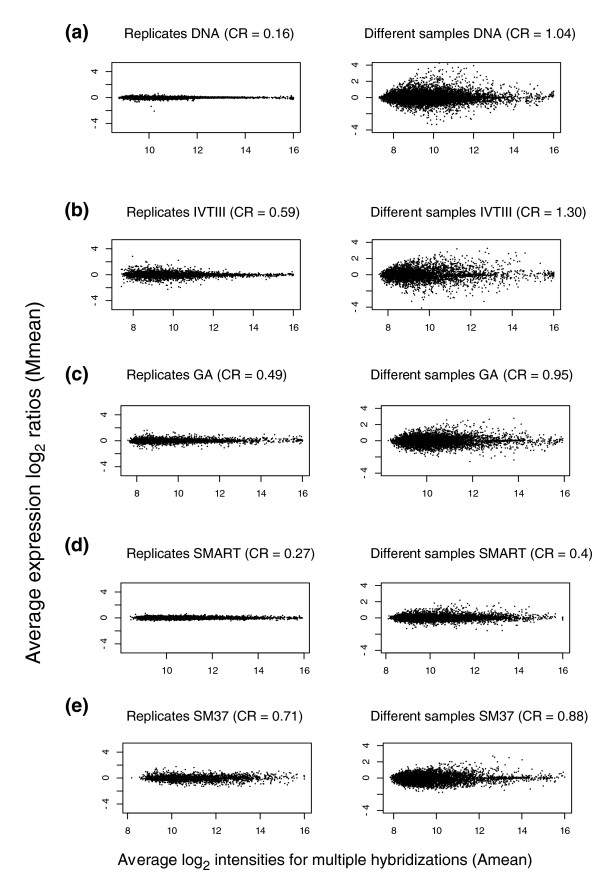
Expression data for unamplified and amplified cDNA demonstrate that all methods introduce errors to microarray expression data. Column 1 represents averaged expression data for dye-swap hybridizations of two technical replicates from the same starting RNA. Column 2 represents averaged expression data for four hybridizations including dye swaps comparing gene expression between two different cell types (3T3 vs OV). **(a) **Unamplified cDNA; **(b) **T7-based amplification; **(c) **global polyadenylated PCR-based amplification technique; **(d) **SMART amplification; **(e) **Modified SMART amplification. CR, coefficient of repeatability, a measure of the repeatability of hybridizations, with lower values representing similar hybridizations (see text for details of calculation).

One approach to testing the reproducibility of an amplification technique is to estimate the levels of variation in expression levels between independently amplified samples. If variation is low, then the amplification technique produces similar products and the reproducibility of amplification is inferred to be high. To minimize the levels of noise, we hybridized on the same slide pairs of technical replicates synthesized from ovarian cell line total RNA by each amplification method or by reverse transcription (unamplified cDNA; Figure 3). The data shown represent the average data from two replicate dye-swap hybridizations.

Amplification of RNA by each method resulted in higher CR values compared with unamplified targets. Nevertheless, SMART-generated replicates demonstrated very low variability between each other, with a CR value (0.27) similar to unamplified DNA (0.16) (Table 1). For GA- and IVTIII-amplified pairs of replicates, CR values were 0.49 and 0.59, respectively, and particularly high variability between replicates was found for SM37-amplified targets (CR = 0.71).

Amplification errors that generate differences between two technical replicates are also reflected in the number of genes calculated as differentially expressed in two hybridizations. Only three outliers were selected for unamplified replicates by a Bayesian method at a *P *value of 0.01 with threshold LOD greater than 0. Surprisingly, SMART-generated replicates did not possess any outliers at all, whereas the other methods of amplification resulted in 75-200 false-positive differentially expressed genes (see below for further discussion of this effect).

### Expression ratios from PCR-amplified cDNA correlate best with those from unamplified cDNA

From the analysis of the errors introduced by amplification reported above, the most reliable RNA amplification technique was found to be SMART PCR-based exponential amplification. To test this further and to analyze the ability of each method to correctly identify differences between RNA samples, we performed a model experiment in which gene expression was compared between two different cell lines.

To do so, single-cell equivalents of total RNA from each cell type were amplified using each method by the strategy outlined above. Gene expression was compared between the two cell types using each method independently. We repeated microarray hybridizations four times for each type of amplification as well as for unamplified cDNA targets, including two independent replicates for each cell line and two dye-swap hybridizations.

**Figure 4:**
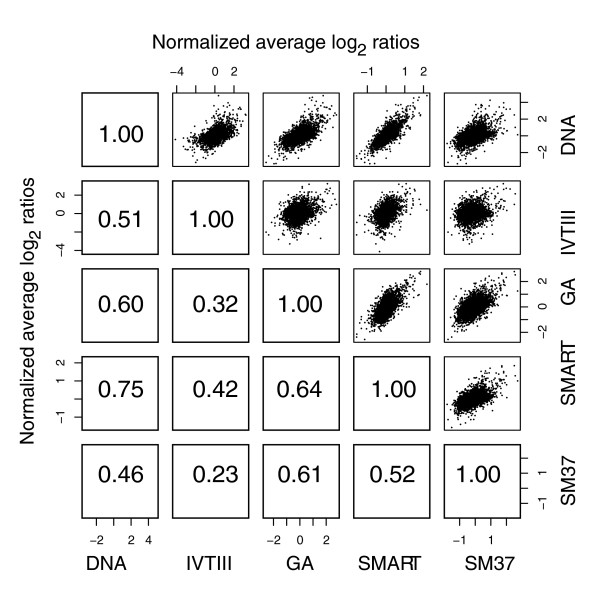
Correlation of data generated by each amplification method with those generated with unamplified cDNA. Matrix of scatterplots showing the relationship between expression ratios comparing the two different cell lines obtained from amplified and unamplified cDNAs generated using each amplification techniques. The *x*- and *y*-axes show virtual *M *values (normalized average log ratios) measured from unamplified or amplified cDNA. Numbers demonstrate Pearson's correlation coefficients for virtual M-values between pairs of each type of hybridizations. DNA, unamplified cDNA; IVTIII, three rounds of linear T7-based amplification; GA, global polyadenylated PCR-based amplification; SMART, SMART PCR-based amplification; SM37, modified SMART PCR-based amplification.

To systematically evaluate the fidelity of the different amplification techniques, we compared Pearson correlation coefficients between the averaged gene-expression ratios for data generated using unamplified cDNA and cDNA generated using each of the amplification methods (Figure 4). The scatterplot matrix and *r*^2 ^values were calculated for the virtual arrays, by which we mean average normalized log(base2) ratio of signal intensities of Cy5 to Cy3 fluorescence across all four hybridizations. The highest correlation coefficient (with data from unamplified samples) was found for SMART-amplified targets (0.75), followed by GA (*r*^2 ^= 0.60), IVTIII (*r*^2 ^= 0.50), and SM37 amplification (*r*^2 ^= 0.43).

### Differential expression discovery rates of each method identify strengths and weaknesses of each approach

**Figure 5:**
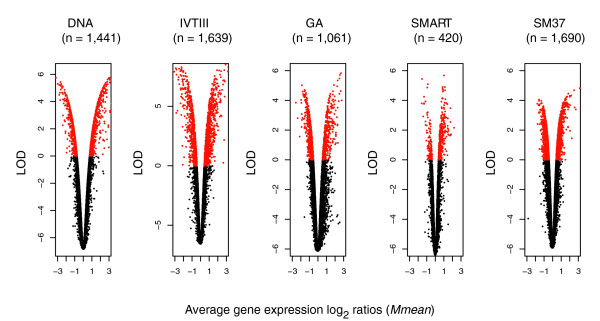
Identification of reproducible, differentially expressed genes from cDNA generated by each amplification method. Volcano plots show LOD for genes across four hybridizations including dye swaps of two different cell samples (OV vs 3T3). Red spots denote differentially expressed genes selected with an empirical Bayesian method with threshold at LOD score zero. The numbers of differentially expressed genes are indicated on each graph (see also Table 2). DNA, unamplified cDNA; IVTIII, three rounds of T7-based amplification; GA, global polyadenylated PCR-based amplification technique; SMART, SMART PCR-based amplification; SM37, modified SMART amplification.

Although SMART-generated expression data correlated best at a global level with data generated using unamplified cDNA, we wished to further investigate the performance of all of the amplification methods in accurately identifying gene expression differences between cell types. To do so, we compared the number of differentially expressed genes selected by a Bayesian method at a *P *value of 0.01 and threshold at LOD = 0 across the all amplifications (Figure 5). Differentially expressed genes that were commonly identified as such in both the tested technique and unamplified cDNA were designated true positives, whereas those that did not match to those identified as differentially expressed in unamplified cDNA were scored as false positives. Those that were differentially expressed in unamplified samples, but were not scored as such in the tested amplification method, were scored as false negatives.

As shown in Figure 5, the highest numbers of differentially expressed genes were identified using IVTIII- and SM37-amplified cDNA and the smallest number were found for SMART amplification (1,639 and 1,690, respectively, for IVTIII and SM37, compared with 420 for SMART). However, the percentage of true positives in the set of genes identified as differentially expressed was much higher for SMART-amplified samples (80%), compared with GA amplification (53.2% true positives), IVTIII (38.9%) and SM37 (33.8%; see Table 2 for details).

Reflecting the overall lower absolute number of genes identified as differentially expressed by SMART, the numbers of the false negatives were slightly lower for all of the other methods (false negative outliers were calculated as DNA outliers minus true positives). Thus all of the methods identify more differentially expressed genes than SMART (compared with data generated from unamplified cDNA), but at the expense of a considerably higher false-positive rate (ranging from 7-12-fold more false-positive genes for those methods).

### SMART PCR-based amplification linearly decreases the expression ratios in amplified samples

Analysis of these data generated an interesting observation on the statistical behavior of microarray data for amplified targets. As expected, the distribution of averaged expression values across hybridizations (*M *values) when comparing gene expression between different cell lines was wider than that observed between replicates and considerably higher than for self-self hybridizations (see Figure 3). Similarly, CR values for virtual arrays (see Table 1) were higher in hybridizations comparing between cell types than in replicate or self-self hybridizations. Thus, whereas the CR value for unamplified cDNA was approximately 1.0, linear T7 amplification resulted in a wider ratio distribution (CR = 1.30), possibly as a result of introducing random noise. GA cDNA demonstrated CR values very close to unamplified samples, but SMART-based amplifications, particularly the standard SMART method, generally decreased variability between two hybridized targets, both in replicates or difference hybridizations (see Table 2).

**Figure 6:**
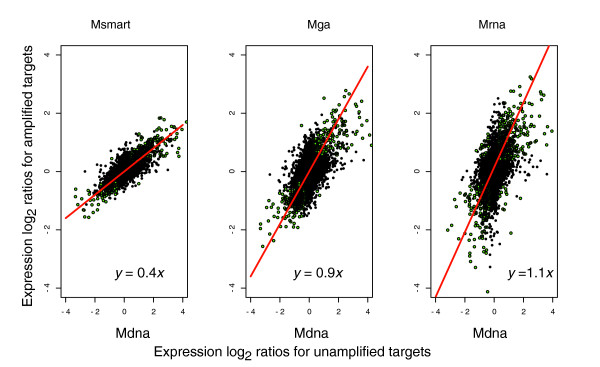
Linear relationships between gene-expression ratios from amplified and unamplified cDNA. SMART-generated cDNA results in systematically reduced expression ratios compared to other methods. Average expression ratios (*M *values) comparing two different cell lines (3T3 vs OV) for unamplified cDNA (Mdna) were plotted against those calculated using cDNA/RNA amplified with each method (Msmart, SMART PCR-based amplification, Mga, global PCR amplification; Mrna, three rounds of T7-based linear isothermal amplification). Green spots indicate differentially expressed genes common to amplified and unamplified samples (see also Table 2). Scatterplot demonstrating the compression effect of SMART amplification: the *M *values of genes for SMART-amplified cDNA are proportionally smaller than those of unamplified cDNA. The compression was found to be linear, fitting the formula *M*_*dna *_= 0.4 *M*_*smart *_(red line). In contrast to SMART, coefficients of linear regression for GA and IVTIII cDNA plotted against unamplified DNA were approximately equal: -0.9 and 1.1, respectively.

SMART amplification systematically reduces gene expression ratios when amplifying microgram amounts of total RNA [23]. Plotting average expression ratios generated using unamplified and SMART-amplified single-cell amounts of RNA clearly demonstrates this effect, such that there is a simple, linear relationship between the expression ratios measured using each approach (Figure 6). In the case reported here, this resulted in a systematic reduction in the gene-expression ratios measured from SMART-amplified material. No significant change was seen in expression ratios generated by GA amplification or by linear isothermal amplification (see Figure 6).

**Figure 7:**
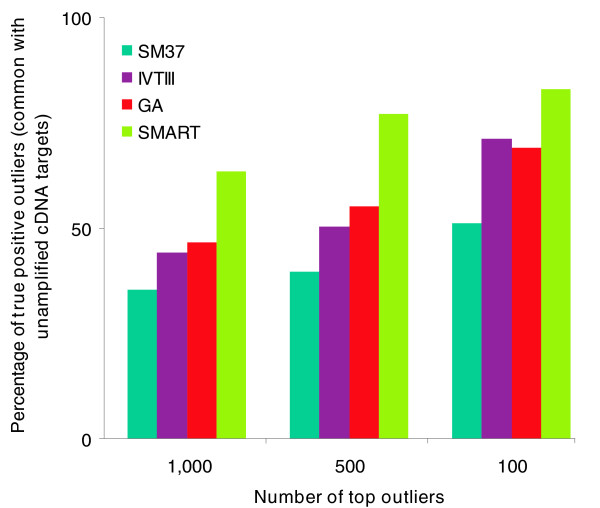
SMART has the highest true-positive rate of differentially expressed genes across a range of empirical thresholds. The outliers for each amplification method were selected for four 3T3 vs OV hybridizations (two replicates and two dye swaps) with cutoff levels as a fixed number of genes with the highest LOD scores: 1,000, 500, and 100. The number of true-positive outliers was calculated by intersecting the set of outliers at each given threshold level for each amplification technique with the set of outliers selected for unamplified targets. The total number of selected outliers for each amplification at a given threshold was designated as 100%. The LOD scores therefore varied depending on cutoff level and type of amplification (see also Table 3). IVTIII, T7-based linear amplification; GA, global polyadenylated PCR amplification; SMART, SMART PCR amplification; SM37, modified SMART amplification.

The reduction in expression ratios observed with SMART (referred to here as a compression effect on observed ratios) would reduce the overall discovery rate of differentially expressed genes when applying a statistical threshold. To compensate for the compression effect in our evaluation of SMART amplification of single-cell amounts of RNA, we calculated the rate of true-positive outliers identified by each method within three fixed thresholds of numbers of differentially expressed genes (the top 100, 500, and 1,000 differentially expressed genes; Table 3). For each amplification technique the LOD values proved to be different depending on the technique and number of top selected outliers. IVTIII was found to have the highest level of LOD score, whereas SMART-amplified samples had the lowest (see Table 3). Replacing the LOD score with empirical cutoffs for numbers of differentially expressed genes, SMART amplification was always found to possess the highest rate of true positives compared with the other three amplification techniques (see Table 3, Figure 7). Thus, regardless of the approach used for identifying differentially expressed genes, SMART consistently had the highest true-positive and lowest false-positive rate of the methods tested.

### SMART and GA amplification performance under real-world conditions

To remove the possible effects of sampling in diluting total RNA to single-cell equivalents and to isolate the effects of amplification on introducing errors into array data, all of the above experiments were carried out under ideal conditions in which the starting material for reverse transcription was 10 ng, the equivalent of 100-1,000 cells. To test whether the findings on the performance of the two PCR-based methods under ideal conditions is predictive of their performance under real-world conditions, we subsequently used each method to amplify total RNA from the two cell lines at a range of concentrations from 1 ng to 10 pg, covering the range of single-cell equivalent amounts of total RNA. As in the experiments described above, cDNA amplified by each method was used to address two questions: the reproducibility of each method (and noise introduced by each method) and the ability of each method to preserve representation of the original starting material, as reflected in their ability to identify true expression differences between the two cell lines.

**Figure 8:**
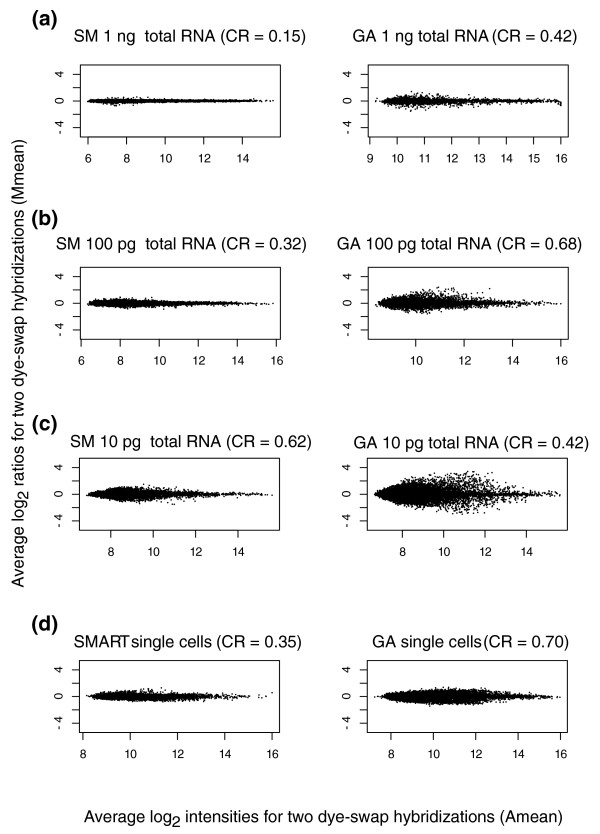
Comparison of expression data generated using each of the PCR-based methods from single-cell equivalents of total RNA and from single neural stem cells. Columns 1 and 2 represent averaged expression data for dye-swap hybridizations of SMART- and GA-amplified technical replicates. Samples of **(a) **1 ng, **(b) **100 pg, and **(c) **10 pg of total RNA were amplified by SMART or by GA in two independent amplifications and replicates were co-hybridized on microarray slides in two dye-swap hybidizations. Total RNA was extracted from a group of 12 cells obtained from developing mouse forebrain (neocortex), mixed together, divided in four equal samples and two samples were independently amplified either by SMART or GA amplification. Technical replicates generated by the same method were co-hybridized on microarray slides in two dye-swap hybridizations. The figures are averaged data for two dye-swap experiments. **(d) **Expression data for single neural stem cells. CR, coefficient of repeatability.

As expected, the CR of each method when comparing independent amplifications from the same starting RNA pool increased with the reduction in amount of input RNA (Figure 8). However, SMART-amplified material consistently had a twofold lower CR at all amounts of input RNA, as it also did in the ideal experiments reported above (see Figure 3), reflecting the lower levels of noise introduced by SMART amplification. Using each method to amplify RNA from pools of two or three murine neural stem cells, freshly isolated from the developing forebrain, resulted in the same finding: comparing independent amplifications, SMART had a twofold lower CR than GA.

Comparing gene expression between the two cell lines at the 10 pg input RNA level, or single-cell equivalent, again generated similar data to the ideal situation. Whereas SMART identified fewer differentially expressed genes, or outliers, compared to GA when applying a fixed probability threshold (239 vs 824; see Table 4), over 70% (169/239) of the genes identified as differentially expressed by SMART were true positives, as assessed by their identification as differentially expressed in unamplified RNA. In contrast, approximately 40% (324/824) of genes predicted as differentially expressed by GA were true positives by the same criterion. Thus, when using a probability cutoff for differential expression, SMART identified fewer truly differentially expressed genes (169 vs 324) but at a higher true-positive rate (70% vs 39%; see Table 4).

Rather than using a probability threshold for differential expression to select the datasets for analysis, we also applied a range of empirical cutoffs for numbers of differentially expressed gene to select the top 100, 500, or 1,000 differentially expressed genes from each dataset for further analysis (Table 5). In this case, SMART had a slightly higher true-positive rate at each cutoff and also identified more differentially expressed genes in absolute number at each cutoff (see Table 5).

## Discussion

### Exponential amplification methods generate reliable data from picogram amounts of RNA

Despite the fact that both linear and exponential RNA amplifications are commonly used methods for expression profiling, little consideration has been given to side-by-side examination of different amplification techniques, particularly for the purpose of single-cell RNA expression profiling. The goal of this study was to test the performance of the most widely used amplification techniques in generating expression data from single-cell amounts of RNA. In addition, we estimated the levels of error for each type of amplification.

Analysis of both technical replicates and test/reference samples hybridized on oligonucleotide microrrays revealed that PCR based-amplifications, and particularly SMART technology, are competitive with, and may outperform, T7-based linear amplification for amplifying picogram amounts of total RNA. We present several findings that demonstrate that PCR amplification generates reproducible microarray data, but with some key shortcomings.

First, PCR-amplified targets possess the least difference between technical replicates among the amplification techniques: CR values for SMART were close to those of unamplified samples (0.27 vs 0.16; see Figure 2), followed by GA and then linear amplification. Secondly, the correlation between unamplified and amplified targets is highest for SMART amplification (*r*^2 ^= 0.75), closely followed by GA (*r*^2 ^= 0.6), with (*r*^2 ^= 0.51; see Figure 4). In addition, the rate of true positives is highest for SMART-amplified cDNA (80%) compared with 53% for GA and 39% for linear amplification. However, a critical difference between SMART and the other methods is the lower overall absolute number of truly differentially expressed genes identified (336 by SMART, as opposed to 492 by GA and 633 by linear amplification).

Extending this approach to analyzing PCR-amplified single cell equivalent amounts of total RNA and also RNA from single neural stem cells found that SMART again outperformed GA in the key areas of CR and true positive rates (see Figure 8, see Tables 4 and 5). SMART also had the lowest overall absolute number of differentially expressed genes, however. We conclude that GA is inherently more noisy than SMART amplification, as reflected in its higher false-discovery rate, but has a higher absolute discovery rate. These results are consistent with previously published findings that exponential amplification methods may yield reproducible results from the picogram range of total RNA [16,18] and be more precise than linear RNA amplification [18,22].

### SMART PCR-based amplification results in compression of microarray expression ratios

It is noteworthy that the distribution of log ratios for SMART-amplified samples is considerably narrower (for both technical replicates and test/reference hybridizations) than for any other method (see Figure 3). We previously observed this compression effect of SMART amplification when amplifying microgram amounts of total RNA [23], finding that it results in a systematic reduction in the magnitude of expression differences between two samples. Therefore, it is likely that the SMART-based technical replicate data appear less noisy than data generated by other methods because of the compression effect on log ratio distribution.

Consistent with our previous findings [23], we found that the decrease in log ratios was also linear when amplifying single-cell amounts of total RNA. In this case, the estimated coefficient of linearity is 2.5. Such a relationship means that the real expression differences between tested samples should be 2^2.5^, or 5.6, times higher than that calculated from the microarray data. No such compression was observed with the other PCR-based amplification method (GA) or with linear isothermal amplification. Global amplification of picogram amounts of total RNA 10^11^-fold has previously been found to substantially increase expression ratios [18], whereas the 10^8^-10^9^-fold GA amplification reported here resulted in a minor change in expression ratios. It is possible that the alteration in ratios seen here with GA amplification could change to the extent of that observed in the work of Iscove and colleagues [18] with the additional 10^3^-fold amplification used in that study.

Although all techniques tested here successfully amplified the starting population of total RNA, we present strong evidence that they also introduce errors to microarray data. Some of the variation is systematic and could be possibly negotiated if reference and test targets are synthesized by the same method. Others are random and could be decreased by replicate hybridizations. In the present investigation we observed that averaging microarray data decreases the values of CR in replicate hybridizations (see Table 1) and increases the correlation between unamplified and amplified targets in test/reference hybridizations by reducing the random component of noise (see Figure 4). The reduction in the contribution of random noise to the false-discovery rate by increasing the number of biological replicate hybridizations could make GA amplification an attractive option for single-cell expression profiling, given the overall higher absolute discovery rate of this method, compared to SMART.

Overall, PCR-amplified samples demonstrate a higher correlation between each other then with T7-amplified targets (see Figure 4), indicating a systematic bias intrinsic to technically similar amplification methods. These data are in good agreement with previous observations of the systematic bias related to the type of hybridization technique which has been demonstrated for both linear and exponential amplifications [28,29].

### Noise in microarray data depend on the rate of RNA amplification

The variability of amplified targets may depend on many factors, including the technical basis of amplification, details of the amplification method and the degree of amplification required. As single cell profiling requires 10^7^-10^9^-fold amplification of the original mRNA population, the number of PCR cycles or number of rounds of linear amplification can become a critical source of errors. Consistent with this, Petalidis and colleagues [22] previously demonstrated a reduction in the discovery rate of differentially expressed genes with numbers of PCR cycles in microarray analysis of SMART-amplified targets when amplifying microgram amounts of total RNA.

For T7-based amplification we also have shown that Pearson's correlation coefficients decreased from *r*^2 ^= 0.90-0.95 for the first round of amplification to *r*^2 ^= 0.7-0.8 for the second round, and finally to *r*^2 ^= 0.5-0.6 for the third round, when amplified targets were correlated with unamplified cDNA (T.S. and F.J.L., unpublished data). One of the sources of variability in T7-amplified samples may be a time-dependent degradation of amplified cRNA that results in shortening of cRNA species [17]. Thus, if each round of linear amplification increases slightly the levels of error, the cumulative effect of three rounds may result in a relatively poor approximation of original mRNA sample.

## Conclusion

The decision as to which amplification technique to use for expression profiling of limiting biological samples depends on several parameters, among them the quality and quantity of RNA, laboratory facilities and the experimental goal. If a goal is to obtain the largest possible number of the differentially expressed genes, GA would be the technique of choice, particularly if the resources are in place to analyze enough cells to reduce the noise in this system. Nanogram amounts of total RNA make it possible to restrict linear amplification to two rounds of T7-based amplification with sufficient yields of labeled targets and high-quality data. Finally, if the rate of true positives is required to be as high as possible, the relatively low false-positive rate of the SMART amplification technique is a useful approach. The decision to use this method should, however, take into account the overall lower number of differentially expressed genes that this approach is likely to identify and also that the real difference in gene expression levels between any two tested samples is likely to be systematically higher than observed using this approach. Further improvements in PCR-based amplification techniques, such as reducing the losses associated with RNA extraction, improved strand switching in the case of SMART, and careful choice of buffers and PCR conditions, may yield even more reproducible results from the picogram range of total RNA.

## Materials and methods

For all experiments, total RNA was isolated from mouse fibroblast (3T3) or mouse ovarian surface epithelium (OV) cell lines using TRI reagent (Amersham Biosciences, Little Chalfont, UK). The ovarian cells were a kind gift of Cristian Brocchieri (University of Cambridge, Department of Oncology & Hutchison/MRC Research Centre). For generating fluorescently labeled cDNA from unamplified RNA, 100 μg of total RNA from 3T3 or OV cell lines was labeled with amino-allyl dUTP during reverse transcription followed by coupling with Cy3 or Cy5 NHS esters ([30]; Cy3 and Cy5 Mono-Reactive Dye Packs, Amersham Biosciences).

### Oligonucleotides

The sequences of the oligonucleotides used for the different amplification technologies were as below:

SM1 5'-AAGCAGTGGTAACAACGCAGAGTAC(T)_30_VN-3'

SM2 5'-AAGCAGTGGTAACAACGCAGAGTACGCrGrGrG-3'

SM37 5'-AGGGAGGCG(T)_24_

SMPCR 5'-AAGCAGTGGTAACAACGCAGAGT-3'

Anchored 5'-TATAGAATTCGCGGCCGCTCGCGA(T)_24_-3'

### SMART cDNA amplification

cDNA synthesis for SMART was performed essentially as described [25]. Total RNA (10 ng) was mixed with 10 pmol of SM1 primer and 10 pmol template-switching SM2 primer in volume of 5 μl. The reaction mixture was incubated at 70°C for 2 minutes and then placed on ice for 2 minutes. The following reagents were then added, 2 μl 5× first strand buffer (Gibco, Carlsbad, USA), 1 μl 20 mM DTT, 1 μl 10 mM dNTPs and 1 μl PowerScript RT (BD Clontech, Mountain View, USA), and the reaction was incubated at 42°C for 1 hour. A 2 μl aliquot of the first strand cDNA was then used for PCR amplification. A 2 μl aliquot of the first-strand cDNA was then used for PCR amplification. The following reagents were added, 80 μl dH_2_O, 10 μl 10× Advantage 2 PCR Buffer (BD Clontech), 2 μl 10 mM dNTPs, 4 μl SMPCR primer and 2 μl 50× Advantage 2 polymerase mix and the reaction mixture was subjected to the cycling program: 95°C for 1 minute and then a variable number of cycles (10 or 28) of 95°C for 15 seconds, 65°C for 30 seconds and 68°C for 6 minutes. cDNA synthesis in a slightly modified SMART technique (SM37) was performed at 37°C rather then at 42°C, and SM1 primer was replaced with primer SM37, followed by PCR amplification with both SMPCR and SM3 primers.

### Global polyadenylated PCR amplification (GA)

10 ng of total RNA (1 μl) was mixed with 3.5 μl of ice-cold stock buffer (25.14 μl DEPC water, 1 μl anchored primer (10 ng/μl), 5 μl 10× reaction buffer (PCR buffer, Roche), 2.5 μl 100 mM DTT, 1 μl 2.5 mM dNTPs, 0.25 μl NP-40, 1 μl RNase inhibitors mix (1:1 mixture rRNasin (Promega, Madison, USA) and Prime (Brinkman/Eppendorf, Hamburg, Germany)) and incubated for 1 minute at 65°C followed by placing on ice for 2 minutes. Then 0.5 μl of RT mix (3 ml PowerScript RT, 0.5 μl of RNase inhibitor mix) was added to RNA and the reaction was incubated at 37°C for 90 minutes. The reaction was stopped by heating to 65°C for 10 minutes and cooled to 4°C. To perform poly(A) tailing of synthesized cDNA 5 μl of TdT mix (0.15 μl 100 mM dATP, 0.5 μl 10× reaction buffer, 0.3 μl 25 mM MgCl2, 3.55 dH_2_O, 0.25 μl TdT (Roche, Lewes, UK), 0.25 μl RNaseH (Roche)) was added to the reaction mixture and the reaction was incubated for 20 minutes at 37°C followed by inactivation at 65°C for 10 minutes. A 2 μl aliquot of the first-strand polyadenylated cDNA was then used for PCR amplification. The following reagents were added: 67 μl dH_2_O, 10 μl 10× Taq PCR Buffer (Takara Bio, Shiga, Japan), 10 μl MgCl2, 2 μl 2.5 mM dNTPs, 2 μl anchored primer (1 μg/μl) and 1 μl LA Taq (Takara Bio) and the reaction mixture was subjected to the cycling program: 95°C for 1 minute, 37°C for 5 minutes, 72°C 20 minutes (once) and then a variable number of cycles (10 or 28) of 95°C for 30 seconds, 67°C for 1 minute and 72°C for 6 minutes. To avoid sampling effects (see Results for further details), 10 ng of total RNA was always taken for cDNA synthesis in all amplifications. This amount is approximately 1,000 times higher then the amount of total RNA in a single cell (around 10 pg). To adjust the amount of RNA to single-cell content, one-fifth of the resulting cDNA was used for first-round PCR. After ten cycles of exponential amplification, 1/200 of the amplified product was taken for a further 28 PCR cycles. When the starting amounts of RNA were l ng, 100 pg, or 10 pg, all of the reverse-transcribed cDNA was used for initial PCR amplification. After ten cycles of PCR, each amplified cDNA was diluted to single-cell equivalents (1 ng starting material was diluted 1/100, 100 pg diluted 1/10), and a second amplification round of 28 PCR cycles was carried out. PCR products were purified with the CyScribe GFX Purification kit (Amersham Biosciences) and indirectly labeled with aminoallyl dNTP using Klenow DNA polymerase (BD Biosciences, Franklin Lakes, USA) followed by coupling with Cy3 or Cy5 NHS esters [30].

Neuronal stem cells were obtained from dissections of developing mouse neocortex at embryonic day 11.5 (E11.5). Dissected neocortices were dissociated with a papain dissociation system (Worthington Biochemical Corporation, Lakewood, USA). Single cells were picked using glass capillary tubes, washed in PBS and placed in PCR tubes. A group of 12 cells were pooled, mixed with 100 ng of polyinositol as a carrier and total RNA was isolated using TRI-reagent (Amersham Biosciences). RNA was dissolved in water and divided into four parts for amplification by SMART or the GA technique (two replicates for both methods).

### T7 based amplification

Linear amplification was performed using the MessengerAmp II aRNA kit (Ambion, Austin, USA). Ten nanograms of total RNA was used for the first-round amplification; 1/10 of the resulting cRNA (cRNAI) was used for the second round of amplification, and then 1/100 of second-round cRNA (cRNAII) was used for the third round of amplification. cRNA was indirectly labeled with amino-allyl-UTP during *in vitro *transcription and coupled with Cy3 or Cy5 NHS-esters as described [31].

### Microarray hybridization

Expression microarrays containing 23,232 65-mer oligonucleotides (Sigma-Genosys, UK) were printed on CodeLink slides (Amersham Biosciences). Hybridized arrays were scanned in an Axon microarray scanner at a resolution of 10 μm at maximum laser power and photomultiplier tube voltage of 60-80%. Image analysis and feature analysis were performed with GenePix Pro 4.0 (Axon Instruments, Foster City, USA).

### Statistical methods

All statistical analysis was conducted using the R environment [32] and the R package Statistics for Microarray Analysis [26]. Log intensity ratios for each spot were obtained with background subtraction. Data normalization was performed using print lowess normalization using the Limma package [33]. Differential genes were identified using an empirical Bayesian method with threshold at LOD score of zero or higher (if specified) [34]. The Pearson correlation coefficient and CR were calculated as described [35].

## Figures and Tables

**Table 1:** Average coefficients of repeatability for two targets hybridized on the same array

Sample	Self-self	Replicates	Different targets
DNA	0.260	0.29 ± 0.03	1.00 ± 0.11
	-	(0.16)*	(1.04)*
IVTIII	0.265 ± 0.004	0.64 ± 0.014	1.24 ± 0.12
	(0.163) *	(0.59)*	(1.29)*
GA	0.287	0.80 ± 0.065	1.03 ± 0.21
	-	(0.49)*	(0.96)*
SMART	-	0.42 ± 0.07	0.60 ± 0.13
	-	(0.27)*	(0.49)*
SM37	-	0.87 ± 0.16	0.94 ± 0.11
	-	(0.71)*	(0.88)*

CR = 2SD(log_2_(*R*)/log_2_(*G*). *CR values for averaged microarray data.

**Table 2:** Summary of expression data obtained by different methods of amplification

Sample	IVTIII	GA	SM37	SMART
Number of outliers	1,639	1,061	1,690	420
True positive	633/1639	492/1,061	572/1,690	336/420
False positive	1,006/1,639	569/1,061	1,116/1,690	84/420
False negative	768/1,441*	949/1,441*	869/1,441*	1,021/1,441*
True positive, percentage of selected	38.6%	53.2%	33.8%	80%

*Number of selected outliers for unamplified targets

**Table 3:** The number and percentage of true-positive outliers of the outliers with the highest LOD score for tested amplification techniques

Cutoff level (top number of outliers)	IVTIII		GA		SMART		SM37	
				
	True positive	LOD score	True positive	LOD score	True positive	LOD score	True positive	LOD score
1,000	442 (44.2%)	1.646	464 (46.4%)	0.156	653 (65.3)	-0.717	353 (35.3%)	0.932
500	251 (50.2%)	3.486	275 (57.0%)	1.412	385 (67.0%)	-0.315	198 (39.6%)	1.850
100	71 (71.0%)	6.300	69 (69.0%)	3.245	85 (85%)	2.110	51 (51.0%)	3.235

**Table 4:** Summary of expression data obtained by SMART and GA amplification techniques applied to 10 pg of total RNA from NIH 3T3 fibroblast cells

Sample	CR		Number of outliers	True positive	False positive	False negative	True positive, percentage of selected
						
	Replicates	Different samples					
GA	1.19	1.62	824	324/824	500/824	1,117/1,441*	39.3%
SMART	0.62	0.54	239	169/239	70/239	1,272/1,441*	70.1%

*Number of selected outliers for unamplified targets.

**Table 5:** Number and percentage of true-positive outliers with the highest LOD score for SMART and GA amplification techniques applied to 10 pg of total RNA from NIH 3T3 fibroblast cells

Cutoff level (top number of outliers)	SMART		GA	
		
	True positive	LOD score	True positive	LOD score
1,000	432 (43.2%)	-3.13	324 (32.4%)	-0.45
500	311 (62.20%)	-1.48	281 (56.0%)	0.315
100	81 (81.0%)	1.43	40 (40%)	2.110
